# Clinicopathological and sonographic characterization of NTRK-fusion papillary thyroid carcinoma based on preoperative molecular testing: a comparative study with BRAF^V600E^ PTC

**DOI:** 10.3389/fonc.2026.1779894

**Published:** 2026-03-05

**Authors:** Yuzhi Zhang, Daoyuan Zou, Xin Wu, Min Han, Junfang Gai, Wenbo Ding, Shuhang Xu, Chao Liu, Xinping Wu, Yuguo Wang

**Affiliations:** 1 Department of Ultrasound, Affiliated Hospital of Integrated Traditional Chinese and Western Medicine, Nanjing University of Chinese Medicine, Nanjing, China; 2 Department of Pathology, Affiliated Hospital of Integrated Traditional Chinese and Western Medicine, Nanjing University of Chinese Medicine, Nanjing, China; 3 Endocrine and Diabetes Center, Affiliated Hospital of Integrated Traditional Chinese and Western Medicine, Nanjing University of Chinese Medicine, Nanjing, China

**Keywords:** BRAF^V600E^ mutation, lymph node metastasis, NTRK fusion, papillary thyroid carcinoma, ultrasonography

## Abstract

**Background:**

NTRK fusions are relatively rare in papillary thyroid carcinoma (PTC), and their clinicopathological characteristics, particularly in unselected populations and in comparison with BRAF^V600E^ PTC, have not been systematically elucidated.

**Methods:**

In this retrospective study, we analyzed PTC patients who underwent surgery between October 2022 and May 2025. All patients underwent preoperative fine-needle aspiration biopsy and multigene molecular testing. Ultimately, 38 patients with NTRK-fusion PTC and 1196 patients with BRAF^V600E^ PTC were included. A comprehensive analysis of the clinical, ultrasonographic, and pathological features of NTRK-fusion PTC was conducted, with comparison to BRAF^V600E^ PTC.

**Results:**

Among the 38 identified NTRK-fusion PTC patients, NTRK3 (81.6%) was the predominant fusion type. Histologically, classical PTC and mixed growth patterns with follicular architecture (34.2% each) were most frequent, followed by the follicular variant (18.4%). NTRK-fusion PTC demonstrated a high rate of lymph node metastasis (LNM) (78.9%). Among preoperative parameters, a tumor diameter >12 mm on ultrasound was associated with increased risk of lateral LNM (OR = 5.00, 95% CI: 1.10-22.82; *P* = 0.038). Besides, NTRK1-fusion PTCs demonstrated a significantly higher frequency of bilateral lobe involvement compared to NTRK3-fusion PTCs (57.1% vs. 12.9%, *P* = 0.025). Compared to patients with BRAF^V600E^ PTC, those with isolated NTRK-fusion (n=34) were significantly younger (median age: 35.0 vs 43.0 years), had larger tumors (median diameter: 10.5 vs 7.0 mm), higher rates of LNM (76.5% vs 50.7%), and greater prevalence of co-existing Hashimoto’s thyroiditis (61.8% vs 28.3%) and follicular nodular disease (26.5% vs 10.6%) (all *P* < 0.01). Cytopathologically, NTRK-fusion PTC demonstrates a higher proportion of atypia of undetermined significance/follicular neoplasm compared to BRAF^V600E^ PTC (41.2% vs. 16.1%). Sonographically, isoechogenicity (20.6% vs. 7.9%), microcalcifications (79.4% vs. 58.0%), and a wider-than-tall shape (91.2% vs. 52.5%) were more frequently observed in the NTRK-fusion group (all *P* < 0.05).

**Conclusions:**

NTRK-fusion defines a distinct PTC molecular subtype characterized by a high burden of LNM and a spectrum of features linked to follicular growth patterns. These findings facilitate the preoperative identification of this tumor subtype and provide a foundation for individualized risk stratification and tailored management strategies.

## 1 Introduction

Thyroid cancer is the most common endocrine malignancy. According to the latest 2022 global statistics, thyroid cancer ranks seventh among all cancers in incidence rate (4.1%), with particularly high rates observed in East Asia (1). Papillary thyroid carcinoma (PTC) is the most prevalent pathological type, accounting for approximately 85% of all thyroid cancers. Although PTC generally carries a favorable prognosis, a subset of patients may develop extensive lymph node metastasis (LNM), local invasion of adjacent structures, or face risks of recurrence and distant metastasis, which can adversely impact both quality of life and overall survival.

Genetic alterations play a critical role in the pathogenesis and progression of thyroid cancer. Molecular testing has been widely adopted in clinical practice for diagnosis, risk estimates, and treatment guidance in thyroid carcinoma (2). The BRAF^V600E^ mutation represents the most prevalent genetic alteration in PTC, with concurrent BRAF and TERT promoter mutations being recognized as a predictor of poor prognosis. In addition to point mutations, gene fusions also contribute significantly to thyroid tumorigenesis. Neurotrophic tropomyosin receptor kinase (NTRK) gene fusions represent the second most common fusion alteration in thyroid cancer, second only to RET. The NTRK family consists of three genes (NTRK1, NTRK2, and NTRK3), which encode for three tyrosine receptor kinase (TRK) proteins (TRKA, TRKB, and TRKC, respectively). These kinases regulate crucial cellular processes through key signaling pathways including MAPK, PI3K/AKT, and PLC-γ (3, 4). NTRK fusions represent the most common oncogenic alteration of NTRK genes, functioning as potent tumorigenic drivers through activation of TRK and subsequent dysregulation of downstream signaling cascades (3). NTRK fusions have been identified across multiple solid tumor types, generally demonstrating low incidence rates except in certain rare malignancies (5–7). In thyroid carcinoma, the overall prevalence of NTRK fusions ranges from 2.2% to 3.1%, while in histologically high-risk, advanced, or radioiodine-refractory cases, the frequency increases to 2.5%-4% (8–10). According to previous literature, the incidence of NTRK fusions in adult patients with PTC mostly ranges from 0.56% to 4.8% (11–16). NTRK fusions show elevated prevalence in pediatric PTC (up to 26%) and are potentially associated with radiation exposure (17–20). Regarding clinicopathological characteristics, a few studies have revealed that NTRK fusion-positive thyroid carcinomas more frequently exhibit a higher propensity for LNM, potentially associated with less favorable prognosis (4, 10, 21). Therapeutically, TRK inhibitors (larotrectinib and entrectinib) have been approved for treating solid tumors harboring NTRK fusions, representing a promising therapeutic approach for advanced thyroid carcinomas, particularly well-differentiated subtypes (9, 22, 23).

Current research on NTRK fusions primarily focuses on high-risk or advanced thyroid carcinomas, with limited investigations specifically in PTC. The clinical and pathological characteristics of unselected PTCs with NTRK fusions remains poorly defined. Furthermore, although both NTRK fusions and BRAF^V600E^ belong to the BRAF-like molecular alteration in PTC (12), differences in their clinicopathological characteristics have not been reported. Therefore, this study aims to systematically summarize the clinical, sonographic, and pathological features of an unselected, consecutive NTRK-fusion PTC cohort and compare them with those of BRAF^V600E^ PTC.

## 2 Material and methods

### 2.1 Patients

We retrospectively reviewed all patients who underwent thyroid surgery with a pathological diagnosis of PTC in the Affiliated Hospital of Integrated Traditional Chinese and Western Medicine, Nanjing University of Chinese Medicine between October 2022 and May 2025. Electronic medical records were retrieved to collect data including demographic characteristics, preoperative ultrasonographic findings, cytopathological and molecular testing results, treatment details, and postoperative pathological outcomes. The inclusion criteria were: (1) patients who underwent initial surgical treatment at our center, and were confirmed as PTC by postoperative pathological examination; (2) availability of complete baseline clinical data; (3) preoperative fine-needle aspiration biopsy (FNAB) with multigene testing performed at our institution; (4) presence of either NTRK fusion or isolated BRAF^V600E^. Exclusion criteria included: (1) FNAB-targeted nodules not confirmed as PTC or with indeterminate pathological diagnosis; (2) coexistence of other types of thyroid malignancy; (3) insufficient or missing ultrasonographic data. This study was approved by the ethics committee of the Affiliated Hospital of Integrated Traditional Chinese and Western Medicine, Nanjing University of Chinese Medicine (No. 2025-LWKY-010) and was conducted following the guidelines of the Declaration of Helsinki.

### 2.2 Pathological and genetic testing

At our institution, patients undergoing FNAB are offered the choice of cytopathological examination alone or in combination with single-gene/multigene molecular testing performed on the aspirated samples. The purpose of genetic testing is to assist in diagnosis. All patients signed informed consent and underwent preoperative ultrasound-guided FNAB using 22-gauge or 23-gauge needles. Three or four passes were made for each nodule. After each aspiration, the specimen was smeared on glass slides and fixed with 95% ethanol for cytological examination, and the needle was washed out with 1ml of 0.9% saline. Cytopathological diagnosis was performed according to the 2023 Bethesda System for Reporting Thyroid Cytopathology (TBSRTC) (24). The wash-out liquid was used for genetic analysis. Next-generation sequencing (NGS) was performed to test a multigene panel encompassing 13 thyroid carcinoma-associated gene mutations (BRAF, HRAS, KRAS, NRAS, RET, TERT, TP53, AKT1, EIF1AX, PIK3CA, DICER1, PTEN and TSHR) and 7 types of gene rearrangements (PAX8/PPARG, ALK, NTRK1, NTRK2, NTRK3, RET and BRAF). The methods and procedures of genetic testing have been comprehensively described in our previous papers (25).

After surgery, all patients received definitive pathological diagnoses according to the 2022 WHO Classification of Thyroid Neoplasms (26). For all cases harboring NTRK fusions, pathological slides were re-reviewed by an experienced thyroid pathologist to reassess the histological subtype.

### 2.3 Ultrasound imaging

A comprehensive preoperative neck ultrasound examination was performed on all enrolled patients. The ultrasound images were obtained mainly by the following machines: Samsung RS80A (Samsung Medison), Aloka ProSound F75 (Hitachi Healthcare), and Fujifilm Arietta 850 (Fujifilm Healthcare). Two experienced radiologists retrospectively reviewed and interpreted the ultrasound findings and reached a consensus when differences appeared. The ultrasound characteristics of thyroid nodules were evaluated according to the Thyroid Ultrasound Reporting Lexicon issued by the American College of Radiology (ACR) (27) and the Chinese Thyroid Imaging Reporting and Data System TIRADS (C-TIRADS) (28). The following ultrasound characteristics were evaluated for each nodule: maximal diameter, composition (solid, predominately solid, predominately cystic), echogenicity (hyper-/isoechoic, hypoechoic, or markedly hypoechoic), shape (wider-than-tall, taller-than-wide), margin (smooth, ill-defined, irregular), calcification/echogenic foci (absent, microcalcifications, macrocalcifications, peripheral/rim calcifications). All target nodules were risk-stratified according to C-TIRADS (28).

### 2.4 Follow-up

Follow-up was conducted every 3 months in the first year after the operation, every 6 months during the next 2 years, and then yearly. We retrieved and recorded the most recent follow-up information for all patients, with data censored through May 2025.

### 2.5 Statistical analysis

Statistical analyses were performed using SPSS statistical software (version 27.0). Continuous variables were described as means ± SD (normal distribution) or medians and 1st-3rd quartiles (Q1-Q3). Continuous variables were analyzed using the two-sample Student’s t-test or Mann-Whitney test, and categorical variables using the two-sided Chi-square (χ^2^) test or Fisher’s exact test. The receiver operating characteristic (ROC) curve analysis was used to evaluate the diagnostic performance of parameters. A value of *P* < 0.05 was considered statistically significant.

## 3 Results

### 3.1 Clinical characteristics of PTC patients harboring NTRK fusions

A total of 38 PTC patients were identified as harboring NTRK fusions. The mean age of the cohort was 37.68 ± 12.67 years (range: 12–62 years), with a median maximum diameter of 10.00 mm (Q1 - Q3: 7.50 - 15.25 mm). Total thyroidectomy was performed in 47.4% of patients, while the remainder underwent lobectomy. During a median follow-up period of 16 months (range: 6–25 months), no distant metastases were observed. One patient underwent reoperation and radioactive iodine therapy for cervical lymph node recurrence detected 3 months after the initial surgery. Another patient, in whom ultrasonography detected a suspicious lymph node in the lateral neck area at the 6-month postoperative follow-up, was managed with active surveillance. No evidence of recurrence or metastasis was found in the remaining patients. The clinical and pathological characteristics of PTC patients with NTRK-fusion are summarized in **Table 1**.

**Table 1:** Clinical characteristics of PTC patients harboring NTRK fusions.

Characteristics	N (%)
Sex	
Male	5 (13.2)
Female	33 (86.8)
Age (years)	
≤ 18	2 (5.3)
> 18 and <55	29 (84.2)
≥ 55	4 (10.5)
Tumor size^a^	
≤ 10mm	20 (52.6)
> 10mm	18 (47.4)
Histological subtype	
Classical	13 (34.2)
Mixture of classical and follicular	10 (26.3)
Follicular	7 (18.4)
Mixture of classical and solid	2 (5.3)
Mixture of follicular and solid	2 (5.3)
Mixture of follicular and clear cell	1 (2.6)
Diffuse sclerosing	1 (2.6)
Hobnail	1 (2.6)
Clear cell	1 (2.6)
Multifocality	
Positive	14 (36.8)
Negative	24 (63.2)
Bilateral involvement	
Positive	8(21.1%)
Negative	30(78.9%)
T stage	
T1	32 (84.2)
T2	5 (13.2)
T3	0 (0.0)
T4	1 (2.6)
N stage	
T0	8 (21.1)
T1a	17(44.7)
T1b	13(34.2)
Venous invasion	
Positive	1 (2.6)
Negative	37 (97.4)
Local invasion	
Negative	22 (57.9)
Capsule invasion	15 (39.5)
Gross ETE	1 (2.6)
Hashimoto's thyroiditis	
Present	21 (55.3)
Absent	17 (44.7)
Follicular nodular disease	
Present	9 (23.7)
Absent	29 (76.3)
TBSRTC category	
I	2 (5.3)
II	0 (0.0)
III	10 (26.3)
IV	4 (10.5)
V	18 (47.4)
VI	4 (10.5)
C-TIRADS	
4B	8 (21.1)
4C	30 (78.9)
Co-alterations	
Negative	34 (89.5)
BRAF^b^	3 (7.9)
TP53^c^	1 (2.6)

^a^, the maximum diameter measured on postoperative histopathology; ^b^, a coexisting TPM3-NTRK1 fusion in one case, RBPMS-NTRK3 fusion in two cases; ^c^, a concurrent ETV6-NTRK3 fusion in one case; C-TIRADS, Chinese Thyroid Imaging Reporting and Data System; ETE, extrathyroidal extension; TBSRTC, the Bethesda System for Reporting Thyroid Cytopathology.

Among patients with bilateral lobe involvement, seven underwent FNAB and molecular testing on nodules from both lobes. Molecular analysis revealed RBPMS-NTRK3 fusions in bilateral tumors from one patient. Of the remaining six patients, contralateral tumors harbored BRAF^V600E^ mutations in four cases, a KRAS mutation in one case, and no detectable genetic alterations in one case. All these contralateral lesions were also pathologically confirmed as PTC postoperatively.

### 3.2 Comparison of clinical and pathological features in PTC patients with NTRK1 versus NTRK3 fusion

Among the 38 enrolled patients, 31 (81.6%) harbored NTRK3 fusions and 7 (18.4%) had NTRK1 fusions. The ETV6-NTRK3 fusion was the most prevalent subtype (n=27, 71.1%), followed by TPR-NTRK1 (n=4, 10.5%), RBPMS-NTRK3 (n=3, 7.9%), TPM3-NTRK1 (n=2, 5.3%), EML4-NTRK3 (n=1, 2.6%), and SQSTM1-NTRK1 (n=1, 2.6%).

The clinical and pathological characteristics of PTC patients with NTRK1 versus NTRK3 fusions are summarized in **Table 2**. Both adolescent patients (2/2) harbored NTRK3 fusions (specifically ETV6-NTRK3 fusion), whereas among the 36 adult patients, NTRK3 and NTRK1 fusions were identified in 29 (80.6%) and 7 (19.4%) cases, respectively. Notably, vascular invasion was identified only in a patient with TPM3-NTRK1 fusion. Bilateral lobe involvement was significantly more frequent in the NTRK1-fusion group (57.1%) compared to the NTRK3-fusion group (12.9%, *P* = 0.025). No significant differences were observed between the two groups regarding age, sex, tumor size, histological subtype, multifocality, local invasion, LNM, co-existing Hashimoto’s thyroiditis (HT) or follicular nodular disease (FND), concomitant other genetic alterations, or TBSRTC and C-TIRADS categories (All *P* > 0.05).

**Table 2:** Comparison of clinical and pathological features in PTC patients with NTRK1 versus NTRK3 fusion.

Characteristics	NTRK1 fusion	NTRK3 fusion	*P*
Sex			1.000
Male	1 (14.3)	4 (12.9)	
Female	6 (85.7)	27 (87.1)	
Age (years)	43.71±13.07	36.32±12.39	0.166
Tumor size (mm)^a^	6.00 (4.00-10.00)	11.00 (8.00-16.00)	0.059
Histological subtype			0.293
Classical	4 (57.1)	9 (29.0)	
Mixed pattern with follicular architecture	3 (42.9)	10 (32.3)	
Follicular	0 (0.0)	7 (22.6)	
Others	0 (0.0)	5 (16.1)	
Bilateral involvement			0.025
Positive	4 (57.1)	4 (12.9)	
Negative	3 (42.9)	27 (87.1)	
Multifocality			0.387
Positive	4 (57.1)	10 (32.3)	
Negative	3 (42.9)	21 (67.7)	
Local invasion			0.736
Negative	5 (71.4)	17 (54.8)	
Capsule invasion	2 (28.6)	13 (41.9)	
Gross ETE	0 (0.0)	1 (3.2)	
T stage			0.641
T1	7 (100.0)	25 (80.6)	
T2	0 (0.0)	5 (16.1)	
T4	0 (0.0)	1 (3.2)	
N stage			0.877
T0	2 (28.6)	6 (19.4)	
T1a	3 (42.9)	14 (45.2)	
T1b	2 (28.6)	11 (35.5)	
Hashimoto's thyroiditis			0.207
Present	2 (28.6)	19 (61.3)	
Absent	5 (71.4)	12 (38.7)	
Follicular nodular disease			1.000
Present	1 (14.3)	8 (25.8)	
Absent	6 (85.7)	23 (74.2)	
Co-alterations			1.000
Present	1 (14.3)	3 (9.7)	
Absent	6 (85.7)	28 (90.3)	
TBSRTC category			0.765
I	0 (0.0)	2 (6.5)	
III	2 (28.6)	8 (25.8)	
IV	0 (0.0)	4 (12.9)	
V	5 (71.4)	13 (41.9)	
VI	0 (0.0)	4 (12.9)	
C-TIRADS			1.000
4B	1 (14.3)	7 (22.6)	
4C	6 (85.7)	24 (77.4)	

^a^, the maximum diameter measured on postoperative histopathology; C-TIRADS, Chinese Thyroid Imaging Reporting and Data System; ETE, extrathyroidal extension; TBSRTC, the Bethesda System for Reporting Thyroid Cytopathology.

### 3.3 Preoperative risk factors for lateral lymph node metastasis in NTRK-fusion PTC

This study investigated the associations between preoperative clinical characteristics, cytopathological diagnosis, sonographic features, and lateral LNM (LLNM) in NTRK-fusion PTC. The maximum tumor diameter on ultrasound was the only parameter demonstrating a significant correlation with LLNM (*P* = 0.027). Patient age, sex, co-alterations, sonographic features, TBSRTC and C-TIRADS categories showed no significant associations with LLNM (all *P* > 0.05; **Table 3**). The area under the ROC curve for predicting LLNM using the maximum diameter was 0.722 (95% CI: 0.561-0.882). A cutoff value of 12 mm yielded the maximum Youden’s index, providing a sensitivity of 76.9% and specificity of 60.0% for predicting LLNM. The incidence of LLNM was significantly higher in tumors with a maximum diameter >12 mm (50.0%, 10/20) compared to those ≤12 mm (16.7%, 3/18; *P* = 0.031). Univariate logistic regression analysis showed that a maximum diameter on ultrasound greater than 12 mm was a risk factor for LLNM, with an odds ratio of 5.00 (95% CI: 1.10-22.82, *P* = 0.038).

**Table 3:** Correlation of clinical characteristics with LLNM in NTRK-fusion PTCs.

Characteristics	LLNM	Non-LLNM	*P*
Sex			0.315
Male	3 (23.1)	2 (8.0)	
Female	10 (76.9)	23 (92.0)	
Age (years)	34.31 ±11.03	39.44 ±13.32	0.241
Tumor size (mm)^a^	16.90 (11.05-21.25)	10.50 (8.00-17.00)	0.027
TBSRTC category			0.180
I	2 (15.4)	0 (0.0)	
III	3 (23.1)	7 (28.0)	
IV	0 (0.0)	4 (16.0)	
V	6 (46.2)	12 (48.0)	
VI	2 (15.4)	2 (8.0)	
Co-alterations			1.000
Present	1 (7.7)	3 (12.0)	
Absent	12 (92.3)	22 (88.0)	
Composition			–
Mixed	0 (0.0)	0 (0.0)	
Solid	13 (100.0)	25 (100.0)	
Echogenicity			0.175
Isoechoic	5 (38.5)	3 (12.0)	
Hypoechoic	7 (53.8)	19 (76.0)	
Markedly hypoechoic	1 (7.7)	3 (12.0)	
Margin			0.342
Smooth	0 (0.0)	0 (0.0)	
Ill-defined	1 (7.7)	0 (0.0)	
Irregular	12 (92.3)	24 (100.0)	
Calcification			0.788
Absent	3 (23.1)	4 (16.0)	
Rim/macro-calcification	0 (0.0)	1 (4.0)	
Microcalcification	10 (76.9)	20 (80.0)	
Shape			0.643
Wider-than-tall	12 (92.3)	21 (84.0)	
Taller-than-wide	1 (7.7)	4 (16.0)	
C-TIRADS			1.000
4B	3 (23.1)	5 (23.1)	
4C	10 (76.9)	20 (80.0)	

^a^, the maximum diameter measured on sonography; C-TIRADS, Chinese Thyroid Imaging Reporting and Data System; LLNM, lateral lymph node metastasis; TBSRTC, the Bethesda system for reporting thyroid cytopathology.

### 3.4 Comparison of clinical and pathological characteristics in PTC patients with NTRK-fusion versus BRAF^V600E^

Among the enrolled participants, 34 harbored isolated NTRK fusions, and 1196 harbored isolated BRAF^V600E^. Patients with isolated NTRK-fusion were significantly younger than those with BRAF^V600E^ (35.00 (29.75-47.25) vs. 43.00 (34.00-52.00) years, *P* = 0.005). Compared to patients with BRAF^V600E^ PTC, the NTRK-fusion cohort exhibited significantly larger tumor size, higher tumor stage, greater propensity for LNM, higher prevalence of co-existing HT and FND, and a greater proportion of category III/IV in TBSRTC classification (all *P* < 0.05, **Table 4**). No significant differences were observed between the two groups regarding sex, bilateral involvement, multifocality, or local invasion (*P* > 0.05, **Table 4**).

**Table 4:** Comparison of clinical and pathological characteristics in PTC patients with NTRK-fusion versus BRAF^V600E^.

	NTRK fusion (n=34)	BRAF^V600E^ (n=1196)	*P*
Age (years)			
≤18	2 (5.9)	5 (0.4)	0.007
>18 and <55	29 (85.3)	968 (80.9)	
≥55	3 (8.8)	223 (18.6)	
Sex			0.074
Female	30 (88.2)	895 (74.8)	
Male	4 (11.8)	301 (25.2)	
Tumor size (mm)^a^	10.50 (6.00-15.25)	7.00 (5.00-10.00)	<0.001
Bilateral			0.557
Yes	7 (20.6)	299 (25.0)	
No	27 (79.4)	897 (75.0)	
Multifocal			0.874
Yes	12 (35.3)	438 (36.6)	
No	22 (64.7)	758 (63.4)	
Local invasion			1.000
Negative	20 (58.8)	681 (56.9)	
Capsule invasion	13 (38.2)	460 (38.5)	
Gross ETE	1 (2.9)	55 (4.6)	
T stage			0.033
T1	29 (85.3)	1111 (92.9)	
T2	4 (11.8)	27 (2.3)	
T3	0 (0.0)	19 (1.6)	
T4	1 (2.9)	39 (3.3)	
N stage			0.001
T0	8 (23.5)	590 (49.3)	
T1a	14 (41.2)	430 (36.0)	
T1b	12 (35.3)	176 (14.7)	
Hashimoto's thyroiditis			<0.001
Positive	21 (61.8)	339 (28.3)	
Negative	13 (38.2)	857 (71.7)	
Follicular nodular disease			0.009
Positive	9 (26.5)	127 (10.6)	
Negative	25 (73.5)	1069 (89.4)	
TBSRTC category			0.002
I	2 (5.9)	127 (10.6)	
II	0 (0.0)	3 (0.3)	
III	10 (29.4)	172 (14.4)	
IV	4 (11.8)	20 (1.7)	
V	14 (41.2)	728 (60.9)	
VI	4 (11.8)	146 (12.2)	

^a^, the maximum diameter measured on postoperative histopathology; ETE, extrathyroidal extension; TBSRTC, the Bethesda System for Reporting Thyroid Cytopathology.

### 3.5 Comparison of sonographic features between NTRK-fusion and BRAF^V600E^ PTCs

**Figure 1:**
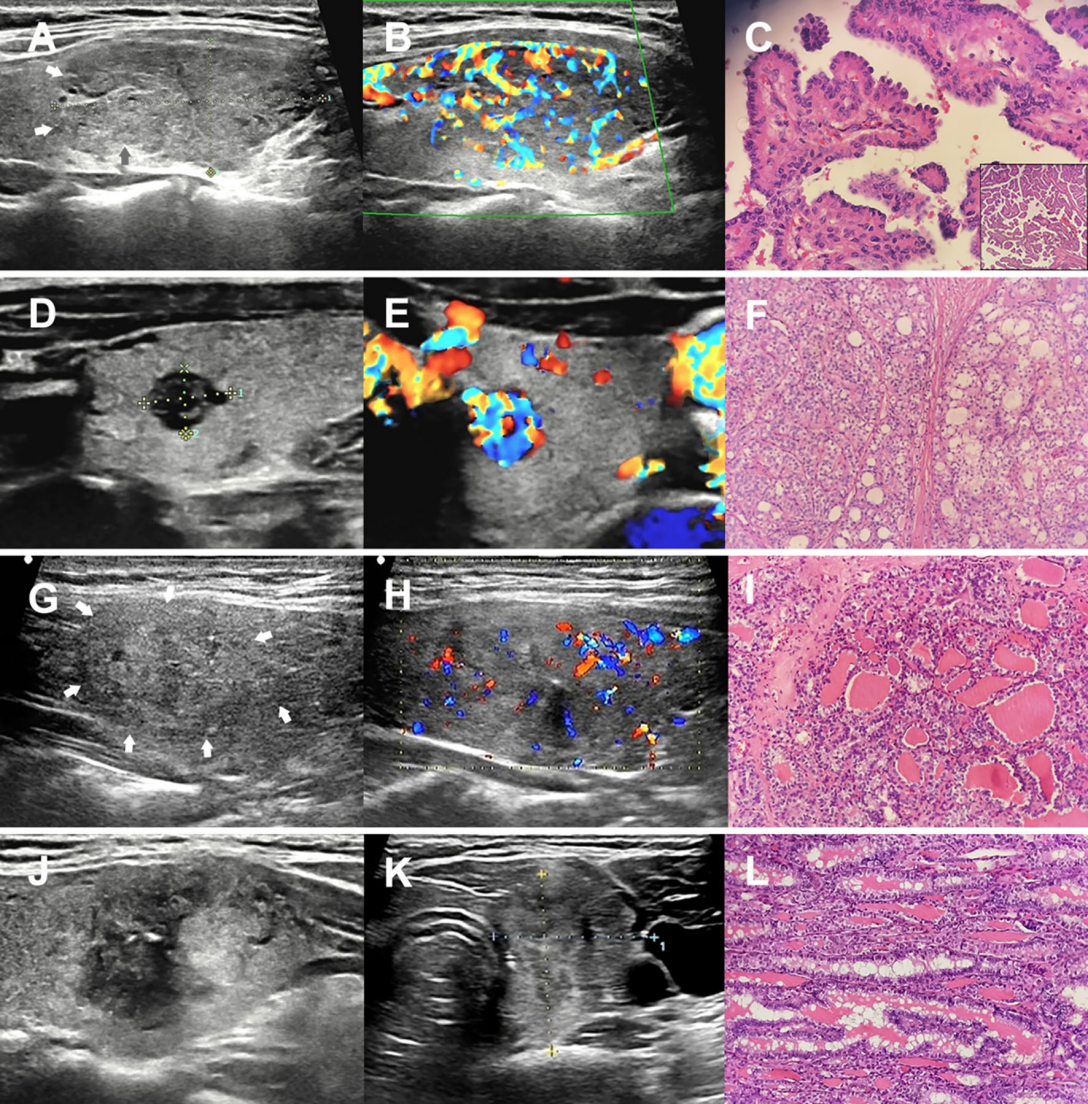
Ultrasonographic and pathological findings of NTRK-fusion PTCs. **(A-C)** a hobnail variant PTC carrying RBPMS-NTRK3 fusion, ultrasonography reveals a mildly hypoechoic solid nodule with irregular margin and rich vascularity. **(D-F)** a PTC harboring SQSTM1-NTRK1 fusion pathologically exhibits a mixed growth pattern comprising solid and follicular structures. Ultrasonographically, it presents as a hypoechoic solid nodule with irregular margin and abundant blood flow. **(G-I)** a follicular variant PTC carrying ETV6-NTRK3 fusion, ultrasonography reveals an isoechoic solid nodule with ill-defined margin, microcalcifications and internal chaotic vascularity. **(J-L)** a PTC harboring TPM3-NTRK1 fusion pathologically exhibits a mixed growth pattern comprising classical and follicular structures. Ultrasonographically, it presents as a solid nodule with mixed hypo- and isoechogenicity, ill-defined margin and microcalcifications.

All NTRK-fusion PTCs exhibited solid composition. Compared to BRAF^V600E^ PTCs, the NTRK-fusion group demonstrated significantly higher frequencies of isoechogenicity, microcalcification and wider-than-tall shape (all *P* < 0.05, **Figure 1**). However, no significant differences were observed between the two groups in other sonographic features or C-TIRADS classifications (*P* > 0.05, **Table 5**).

**Table 5:** Sonographic features of NTRK-fusion and BRAF^V600E^ PTCs.

Sonographic features	NTRK fusion (n=34)	BRAF^V600E^ (n=1196)	*P*
Composition			0.630
Mixed	0 (0.0)	44 (3.7)	
Solid	34 (100.0)	1152 (96.3)	
Echogenicity			0.017
Isoechoic	7 (20.6)	94 (7.9)	
Hypo-/ markedly hypoechoic	27 (79.4)	1102 (92.1)	
Margin			0.357
Smooth	0 (0.0)	69 (5.8)	
Ill-defined	1 (2.9)	79 (6.6)	
Irregular	33 (97.1)	1048 (87.6)	
Calcification			0.047
Absent	6 (17.6)	422 (35.3)	
Rim/macro-calcification	1 (2.9)	80 (6.7)	
Microcalcification	27 (79.4)	694 (58.0)	
Shape			<0.001
Wider-than-tall	31 (91.2)	628 (52.5)	
Taller-than-wide	3 (9.7)	568 (47.5)	
C-TIRADS			0.920
3	0 (0.0)	4 (0.3)	
4A	0 (0.0)	37 (3.1)	
4B	7 (20.6)	246 (20.6)	
4C	27 (79.4)	897 (75.1)	
5	0 (0.0)	12 (1.0)	

C-TIRADS, Chinese Thyroid Imaging Reporting and Data System.

## 4 Discussion

The oncogenic role of NTRK fusions in thyroid tumorigenesis is widely recognized, however, their clinicopathological characteristics in unselected PTC cohorts remain incompletely characterized. Our study systematically profiled the clinical, sonographic, and pathological features of NTRK-fusion PTC (defined by preoperative molecular testing) in comparison with BRAF^V600E^ PTC. Our findings revealed that NTRK-fusion PTC was characterized by a remarkably high rate of LNM, a propensity for follicular growth pattern and atypical features. We also identified tumor size as a preoperative risk factor for LLNM. These findings established NTRK-fusion PTC as a distinct clinicopathologic entity with aggressive tendencies, providing a crucial evidence base for optimizing its preoperative diagnosis and clinical management.

In thyroid cancer, NTRK fusions primarily involve the NTRK1 and NTRK3 genes. Throughout the literature, only a few cases of NTRK2 fusion have been reported (29, 30). Previous studies have identified a variety of fusion partners for NTRK genes, including TPM3, TPR, IRF2BP2, TFG, SQSTM1, TRIM33, NFASC, DIAPH1, LMNA, VCL, EZR, TP53, and PRDX1 for NTRK1 (4, 10, 23, 31, 32), as well as ETV6, EML4, SQSTM1, RBPMS, VIM, and TRIM37 for NTRK3 (10, 23, 32). Our study identified three distinct fusion partners each for NTRK1 (TPR, TPM3, SQSTM1) and NTRK3 (ETV6, RBPMS, EML4), respectively. Most previous studies suggested that NTRK fusions typically do not co-occur with other oncogenic driver alterations. However, a study from the Middle East found that 10.5% (2/19) of NTRK-fusion PTCs harbored a concurrent BRAF mutation (16). Aligning with this finding, our study also observed a relatively high rate of concurrent BRAF^V600E^ in NTRK-fusion PTCs (7.9%). Furthermore, one case with a TP53 co-alteration was identified. We found no significant difference in the prevalence of co-alterations between NTRK1 and NTRK3 fusion cases, and the presence of co-alterations did not significantly increase the risk of LLNM in NTRK-fusion PTCs. Nevertheless, given the limited number of such cases, the clinical implications of co-alterations in NTRK-fusion PTC should be further interpreted with caution and confirmed in larger-scale studies.

Previous studies have indicated that kinase gene fusion was associated with younger age at diagnosis and larger tumor size (15, 33), and the findings of this study support this viewpoint. In this study, NTRK-fusion PTC patients were relatively young, with 84.2% under 55 years of age. The median age of PTC patients harboring isolated NTRK fusion was 35.00 years, markedly lower than that of patients with BRAF^V600E^ (43.00 years). Corroborating this, the proportion of pediatric and adolescent patients was significantly larger in the NTRK-fusion group (5.9%) than in the BRAF^V600E^ group (0.4%). This finding is consistent with the literature review by Tondi Resta et al., which reported that the age or median age of patients with NTRK-fusion thyroid cancer was under 42 years in nearly all studies (32). Although the overall tumor size in this study was relatively small, the median maximum diameter of PTC with isolated NTRK fusion (10.50mm) was still significantly larger than that of BRAF^V600E^ PTC (7.00mm), and the proportion of T2-stage cases in the former group (11.8%) was approximately five times that of the latter group (2.3%).

While most studies reported an association between NTRK fusions and a follicular growth pattern in thyroid cancer, the reported prevalence of this morphology varies considerably across cohorts. Several researchers have found that NTRK-fusion PTC predominantly exhibits mixed growth patterns comprising follicular architecture (41% - 90.9%) (14, 23, 34), whereas the pure follicular variant is less uncommon (17%) (23). In contrast, Tondi Resta et al. (32) identified 9 follicular variant PTC (fvPTC, 40.9%) and 7 classical PTC (cPTC, 31.8%) among 22 NTRK-fusion PTCs. In addition, Ricarte-Filho et al. (35) reported a series of 20 pediatric PTC patients, identifying the fvPTC in 20%, the diffuse sclerosing variant in 10%, and the cPTC in the remainder. Occasional clear cell change has also been documented (14, 34). These discrepancies are likely attributable to differences in patient cohort selection, genetic backgrounds, and pathological diagnostic criteria. In the present study, all NTRK-fusion PTCs were re-evaluated according to the 2022 WHO classification of thyroid tumors (26). We found that cPTC and mixed patterns with follicular architecture were equally prevalent (34.2% each), representing the most common histological subtypes. The fvPTC accounted for 18.4%, with additional cases of diffuse sclerosing, clear cell, and solid-containing mixed patterns also identified. Furthermore, we reported a novel case of hobnail variant PTC harboring an RBPMS-NTRK3 fusion.

Our study found no significant differences in multifocality (35.3% vs. 36.6%) or bilateral involvement (20.6% vs. 25.0%) between isolated NTRK-fusion PTC and BRAF^V600E^ PTC. Previous large-cohort studies have identified BRAF^V600E^ as a risk factor for increased multifocality and bilateral involvement (36, 37). However, whether NTRK fusions independently confer a similar risk requires further investigation in larger cohorts. Notably, in our cohort, molecular analysis of contralateral lobe tumors revealed a higher prevalence of BRAF^V600E^ (4/7) than NTRK fusions (1/7), suggesting that NTRK fusions are less likely to be the primary driver of bilateral involvement.

The existing literature generally agrees that gross ETE is uncommon in NTRK-fusion PTC, however, the reported rates of microscopic ETE vary considerably. The overall rate of ETE in NTRK-fusion PTCs in our cohort was approximately 42.1%, with only one case of gross ETE, a finding consistent with the results reported by Wu et al. (29). Lee et al. (14) observed microscopic ETE in 63.6% (7/11) of their NTRK-fusion cases. In the study by Tondi Resta et al. (32), capsular invasion was identified in 40.9% of cases (9/21), perineural invasion in two (9.1%), and ETE in six cases (27.3%), with one case demonstrating gross ETE. These discrepancies are likely attributable to differences in cohort selection and tumor staging, as the tumors in the latter two studies were larger than those in our cohort. While previous large-scale studies have established BRAF^V600E^ as an independent risk factor for ETE (36, 37), the ETE profiles of NTRK-fusion and BRAF^V600E^ PTCs were remarkably similar in our study. Future investigations are needed to determine whether NTRK fusions independently increase the risk of ETE.

Our study identified a high risk of LNM (78.9%) as a hallmark of NTRK-fusion PTC, thereby confirming previous reports that kinase gene fusions are associated with LNM in this cancer (15, 32). The rate of LLNM in PTC with isolated NTRK fusion (35.3%) was significantly higher than that in BRAF^V600E^ PTC (14.7%). In previous studies on adult PTC, the reported overall LNM rates for NTRK-fusion PTC ranged from 40.9% to 83.9% (14, 29, 32, 34), with a LLNM rate of 30.0% (34). In progressive or histologically high-risk PTC, LNM rates reached 78.3% - 100%, with LLNM rates of approximately 65% (10, 23). According to literature reports, pediatric PTC showed even higher rates of overall (80% - 100%) and lateral (70% - 100%) LNM (23, 35). As our cohort consisted almost entirely of adults, our findings are consistent with previous data in adult. Furthermore, no previous studies have explored risk factors for LLNM in NTRK-fusion PTC. We investigated preoperative clinical characteristics and found that only a maximum tumor diameter greater than 12 mm on ultrasound was associated with an increased risk of LLNM. These results suggest that for patients with suspected PTC harboring NTRK fusions, particularly those with tumor diameter >12 mm on ultrasound, a more thorough preoperative evaluation of cervical lymph node should be performed to guide surgical planning.

In this study, none of the patients presented with distant metastasis at initial diagnosis or during follow-up. Previous literature has reported a relatively high risk of distant metastasis in NTRK-fusion PTC, ranging from 4.5% to 11.1% (14, 16, 32, 34). However, these studies were limited by small sample sizes and included tumors with significantly larger dimensions or more advanced primary T stages compared to the present research. Other studies have shown that in progressive or histologically high-risk NTRK-fusion PTC, the risk of distant metastasis is higher, exceeding 50% (10, 23). The study population in this study consisted of an unselected PTC cohort, generally characterized by smaller tumor sizes and lower T stages, which may explain the discrepancy in findings. In the future, we will continue to follow up with the participants to observe the long-term prognosis of NTRK-fusion PTC.

Few studies have explored the sonographic features and cytopathological characteristics of NTRK-fusion PTC. In terms of ultrasonography, our study found that all NTRK fusion PTCs presented as intermediate to high risk (C-TIRADS 4B - 4C) on ultrasound. Lee et al. (14) summarized the sonographic features of 11 cases of NTRK-fusion PTC and reported that all NTRK-rearranged PTCs were classified as high suspicion. The most common ultrasonic features of NTRK-fusion PTC were solid composition (100%), hypoechogenicity (100%), parallel orientation (63.6%), ill-defined margin (54.5%), and calcification (81.8%). Similarly, all lesions in our study were solid (100%) and shared a similarly high rate of calcification (81.6%). However, differences were observed: our cohort showed higher proportions of isoechogenicity (21.1%, 8/38), irregular margins (97.4%, 37/38), and wider-than-tall shape (86.8%, 33/38). NTRK-fusion PTC diverged from BRAF^V600E^ PTC by demonstrating a higher prevalence of both atypical features (isoechogenicity: 20.6% vs. 7.9%, wider-than-tall shape: 91.2% vs. 52.5%, potentially reflecting follicular growth architecture) and the classic feature microcalcifications(79.4% vs. 58.0%). This paradoxical combination of patterns collectively creates a more complex and heterogeneous imaging profile (**Figure 1**). Regarding cytopathology, a previous study reported that most NTRK-fusion PTC cases were classified as TBSRTC category V (52.4%) (32), while another study found that NTRK-fusion PTC cases were mostly categorized as “malignant” (4/6), with the remainder as “follicular lesion of undetermined significance” (34). In our study, the majority of NTRK-fusion PTC cases were also classified as TBSRTC category V (45.7%), followed by category III (28.6%). Compared to BRAF^V600E^ PTC, NTRK-fusion PTC showed higher proportions of TBSRTC categories III and IV (29.4% vs. 14.4%, 11.8% vs. 1.7%, respectively). We attribute this to the distinct histopathological profile of NTRK-fusion PTC, specifically, the more frequent presence of follicular growth patterns. In summary, compared to BRAF^V600E^ PTC, NTRK-fusion PTC more frequently presents with complex or atypical features on both ultrasonography and cytopathology.

The differences in clinical and pathological characteristics between NTRK1-fusion PTC and NTRK3-fusion PTC remain poorly characterized. A study from the Czech Republic found that NTRK1-fusion thyroid carcinomas demonstrated a higher frequency of multifocality and aggressive features compared to NTRK3-fusion carcinomas (21). Another study from the United States reported that PTC patients with NTRK1-fusion tended to be younger and have a higher incidence of lymphovascular invasion compared to those with NTRK3 fusions in adult patients, although these differences were not statistically significant (23). In contrast, a study from China found that NTRK3-fusion PTCs had slightly higher rates of capsular invasion and LNM compared to NTRK1-fusions (29). While our findings partially align with the latter report, we notably observed trends in NTRK3-fusion PTCs toward younger patient age and larger tumor size, albeit without statistical significance. In addition, our results revealed that NTRK1-fusion PTCs had a significantly higher rate of bilateral involvement compared to NTRK3-fusion PTCs (57.1% vs. 12.9%). The heterogeneity of findings across studies may be partially attributable to the limited sample sizes in each cohorts, underscoring the need for larger studies to further evaluate the differences in clinicopathological features between NTRK1 and NTRK3 fusion PTCs. Furthermore, it is noteworthy that whether the discrepancies in these results are related to ethnic variations remains to be investigated.

Our study has some limitations. First, the statistical power is limited by the small sample size, a challenge inherent in studying the rare NTRK-fusion cases. Future studies with larger cohorts are needed to validate our findings. Second, the comparison of pathological subtypes and prognosis between the BRAF^V600E^ and NTRK-fusion cohorts was precluded by challenges in comprehensive pathological re-review of the large BRAF^V600E^ cohort and its high rate of loss to follow-up. Besides, the follow-up duration was relatively short. Continued monitoring is necessary to evaluate the long-term outcomes of NTRK-fusion PTC, including recurrence and metastasis.

## 5 Conclusions

In summary, this study delineates the distinct clinicopathologic profile of NTRK-fusion PTC through preoperative molecular profiling of an unselected cohort. Key characteristics include a high rate of LNM, frequent follicular-patterned histology, and complex or atypical sonographic/cytopathologic features. Tumor size >12 mm on ultrasound may represent a risk factor for LLNM, and NTRK1 fusions are associated with an elevated risk of bilateral lobe involvement. Compared to patients with BRAF^V600E^ PTC, those harboring NTRK fusions were significantly younger, presented with larger tumor size, higher N stages, and higher rates of co-existing HT and FND. A comprehensive understanding of the clinical profile of NTRK-fusion PTC enables the recognition of this distinct subtype, which is critical for informing preoperative risk stratification and guiding tailored management strategies.

## Data Availability

The original contributions presented in the study are included in the article/supplementary material. Further inquiries can be directed to the corresponding authors.
